# General Purpose Transistor Characterized as Dosimetry Sensor of Proton Beams

**DOI:** 10.3390/s23073771

**Published:** 2023-04-06

**Authors:** J. A. Moreno-Pérez, I. Ruiz-García, P. Martín-Holgado, A. Romero-Maestre, M. Anguiano, R. Vila, M. A. Carvajal

**Affiliations:** 1 ECSens, Imuds, Department of Electronics and Computer Technology, ETSIIT, University of Granada, 18014 Granada, Spain; juanantoniomp@ugr.es (J.A.M.-P.); isirg@ugr.es (I.R.-G.); 2 National Accelerator Center (University of Sevilla, CSIC, JA), 41092 Sevilla, Spain; pmartinholgado@us.es (P.M.-H.); mrmaestre@us.es (A.R.-M.); 3 Department of Atomic, Molecular and Nuclear Physics, University of Granada, Institute for Biosanitary Research, Ibs. Granada, 18012 Granada, Spain; mangui@ugr.es; 4 National Fusion Laboratory, EURATOM-CIEMAT, 28040 Madrid, Spain; rafael.vila@ciemat.es

**Keywords:** general purpose MOSFET, proton beams, dosimetry

## Abstract

A commercial pMOS transistor (MOSFET), 3N163 from Vishay (USA), has been characterized as a low-energy proton beam dosimeter. The top of the samples’ housing has been removed to guarantee that protons reached the sensitive area, that is, the silicon die. Irradiations took place at the National Accelerator Centre (Seville, Spain). During irradiations, the transistors were biased to improve the sensitivity, and the silicon temperature was monitored activating the parasitic diode of the MOSFET. Bias voltages of 0, 1, 5, and 10 V were applied to four sets of three transistors, obtaining an averaged sensitivity that was linearly dependent on this voltage. In addition, the short-fading effect was studied, and the uncertainty of this effect was obtained. The bias voltage that provided an acceptable sensitivity, (11.4 ± 0.9) mV/Gy, minimizing the uncertainty due to the fading effect (−0.09 ± 0.11) Gy was 1 V for a total absorbed dose of 40 Gy. Therefore, this off-the-shelf electronic device presents promising characteristics as a dosimeter sensor for proton beams.

## 1. Introduction

In the last years, proton therapy has become a real alternative to conventional high-energy photon beam therapy and shows up as an increasingly common treatment tool in radiotherapy centers [1,2]. This is mainly due to modern delivery techniques [3,4] and treatment planning strategies [5]. One of the main differences with photon beams is that the proton ones are characterized by the Bragg peak, a markedly high peak that permits a high spatial accuracy in the dose delivery and, consequently, a larger tumor control with a simultaneous sparing of surrounding normal tissues [1,6]. For example, pencil-beam scanning (PBS), one of the most modern treatment modalities used in proton therapy, uses a narrow proton beam that allows spot-by-spot and layer-by-layer dose delivery, providing accurate and complete irradiation of the whole target tumor [7].

As in electron and photon conventional radiotherapy techniques, one of the key points to ensure treatment effectiveness concerns the accuracy of the dose distribution within the irradiated volume. According to ICRU [8], the relative uncertainties in the absorbed dose distributions must be below 5% in the case of external beam radiotherapy. The dosimetry devices that are most commonly employed to measure the dose distributions in water and air are ionization chambers. However, to obtain the actual doses, the corresponding chamber outputs must be corrected by using different factors that, in general, are rather difficult to measure. Traditionally they have been estimated by using Monte Carlo techniques [9].

Other dosimeters, such as semiconductor-based systems [10], have been increasingly used in clinical practice. Two of the main advantages of these devices are their small size and repeatability. Detectors with small active volumes are essential to develop new techniques such as, for example, stereotactic proton therapy where extremely small radiation fields may be involved. Several commercial dosimetry systems, based on diodes or metal-oxide semiconductor field-effect transistors (MOSFETs), are available as radiation sensors [11]. MOSFETs have the advantage of operating at a lower voltage than diodes, and their cost is smaller. Since around 1980, MOSFETs have been tested in proton beam dosimeter applications [12,13]. The usual dosimetric parameter in the corresponding measurements is the voltage threshold shift before and after the irradiation, ΔV_T_, produced by the radiation-induced trapped charge in the gate oxide of the MOSFETs. This electric feature is read out at a constant drain current in the region of minimum thermal drift to reduce the temperature effects in the dose determination. To increase the sensitivity, thick gate oxide is fabricated under special conditions; these MOSFETs are so called RAFETs (RADiation Field Effect Transistors) [14]. In fact RADFETs, manufactured by Tyndall Institute (Cork, Ireland) have been successfully tested as proton dosimeters [15]. To assess the MOSFET reliability as dosimeter for proton beams, different dependences (reproducibility, linearity, fading effect, beam intensity, energy, and angular dependence) must be characterized. The commercial system manufactured by Best Medical (Canada) uses the RADFET model TN502RD, that is one of the devices most widely used for patient dose measurements and it has been characterized as proton dosimeter [16], and for in-vivo dosimetry applications [17].

The use of general-purpose commercial transistors as gamma detectors has been extended in the last years [18,19]. The main advantage that the MOSFET that is not manufactured for radiation measurements presents is the low cost (compared to RADFET) and availability. However, due to their low sensitivity, higher amplification and thermal compensation techniques should be applied to reach an acceptable performance to be used as dosimeters in radiotherapy treatment control. Different readout techniques have been successfully applied to commercial transistors to enhance the linearity and reduce thermal effects [20,21,22]. These techniques have made it possible for some commercial MOS transistors to be used as clinical dosimeters in radiotherapy. Applying these techniques too, it would be possible to use a commercial transistor as a proton dosimeter as well, and this is the aim of the present work. A dosimeter for proton beams based on general purpose devices, that are not aimed to radiation measurements, would reduce the cost of quality control of the radiation source and the in vivo dosimetry in radiotherapy treatments. The 3N163 from Vishay (USA) has presented very acceptable sensitivity for dose measurements for photo beams at different energies [18,20]. In the present work, the characterization of the 3N163 as proton dosimetry was undertaken under different gate biases.

## 2. Materials and Methods

**Figure 1:**
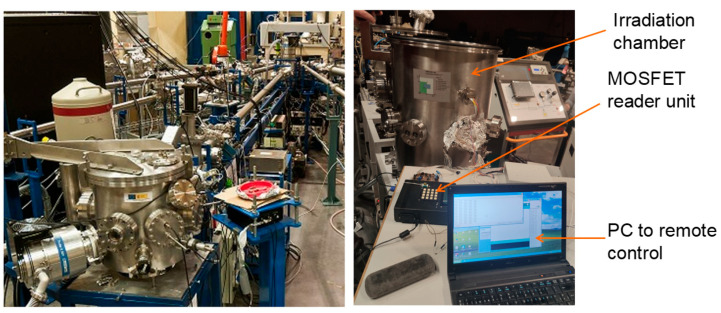
Tandem accelerator (**left**), and experimental setup showing the irradiation chamber, the reader unit, and the control computer (**right**).

The irradiations were carried out at the National Accelerator Centre, CNA (Sevilla, Spain), using the Tandem Accelerator for generating a low-energy proton beam (see Figure 1). It has several beamlines, but only the implantation-irradiation line was used for this test campaign, with protons of energy 5.36 MeV. Since low-energy protons have a very short range in matter, all the irradiation tests were performed in a vacuum chamber (~10^−6^ mbar) placed at the end of the beamline.

Regarding the methodology, we used an approach in line with [23]. First, the proton beam was focused on a scintillator that was placed in the same sample holder as the device under test (DUT), to check the position, shape, and size of the beam to work with around 1 cm^2^ spot size; this pencil area completely covers the sensitive area of the DUT. Second, we measured the proton beam flux by using a Brookhaven 1000c current integrator connected to the sample holder, which was electrically insulated from the rest of the line. Then, we used the 2D beam-rastering system to cover a larger surface with the same conditions, so we reduced the flux to the needed value. Finally, we moved the sample holder to align the DUT with the beam, and we started the irradiation of the DUT with the fixed conditions. The flux was also measured while the beam was sweeping thanks to the Faraday cup configuration of the sample holder.

The fluence associated with each irradiation step was calculated by multiplying the averaged measured flux by the irradiation time. On the other hand, the dose was obtained from the fluence and the value of the stopping power for protons of 5.36 MeV in silicon, that is (55.80 ± 0.01) keV·cm^2^/mg [24].

**Figure 2:**
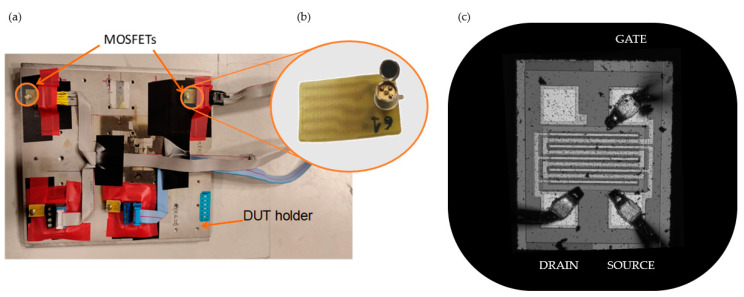
(**a**) Two MOSFET samples placed on the holder. (**b**) Detail of the housing modification showing the silicon die of the MOSFET. (**c**) Silicon die image obtained with the profilometer SNeox.

Inside the irradiation chamber, the DUT was fixed on the holder, as Figure 2a shows. Due to the low penetration depth in matter of the proton beams of this energy (lower than 6 MeV), the top of the device housing was removed (see Figure 2b). In this way, the active volume of the MOSFET, its gate oxide, can be reached by the proton beam. 

The surface morphology study of the MOSFET was carried out with the 3D optical profilometer SNeox (Sensofar, Barcelona, Spain). The confocal technique was configured with ×5 and ×20 optical objectives achieving an optical resolution from 470 to 250 nm (see Figure 2c). The silicon die images were processed by the profilometer software SensoVIEW 2.0.0. With this equipment, channel dimensions were measured, obtaining a width 1.0 mm and a length of 10 µm.

Two MOSFET samples were introduced into the irradiation chamber at the same time but irradiated one by one. Each sample was externally connected with an independent cable with the outside of the ionization chamber via a cable gland. The reader unit was placed outside but close to the ionization chamber. Therefore to change the sample being tested, just the connected cable should be switched. A total of four sets of three samples were characterized, with different bias voltages during irradiation. Each set of samples was biased to 0 V, 1 V, 5 V, and 10 V, respectively.

**Figure 3:**
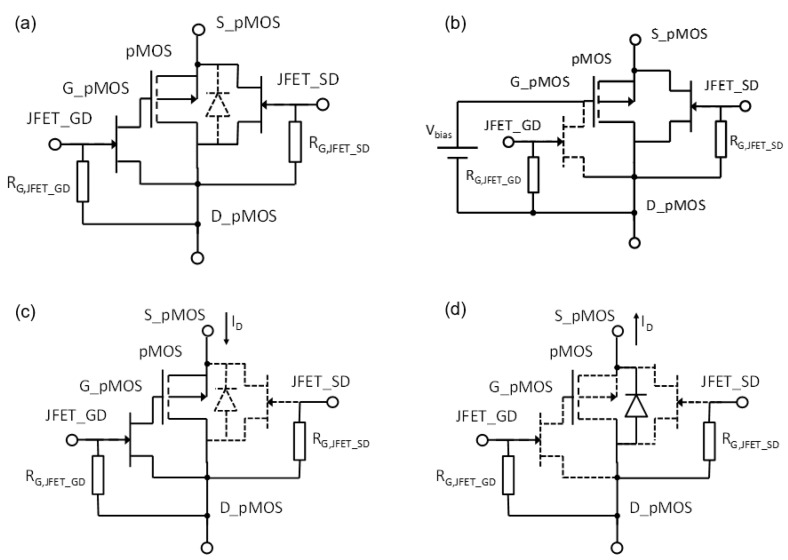
Sensor module configurations at different states: (**a**) Storage mode. (**b**) Sensing mode. (**c**) Readout of V_S_. (**d**) Readout of V_F_.

**Figure 4:**
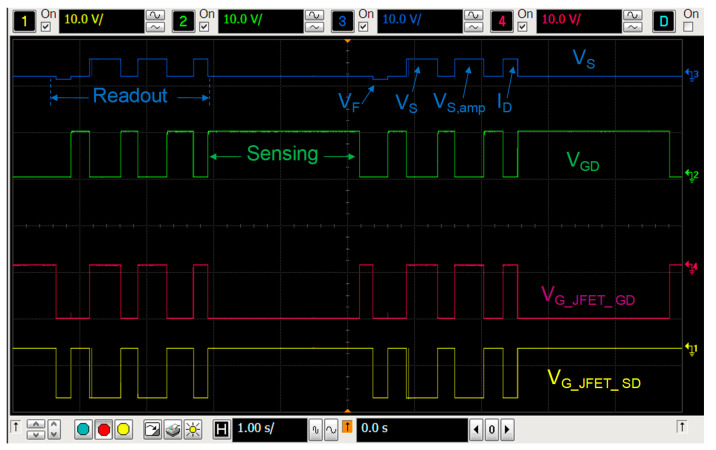
Measurement chronogram: In blue the source voltage is displayed; in green the gate voltage of the MOSFET; in red the gate voltage of the JFET_GD and, in yellow, the gate voltage of the JFET_SD.

The reader unit used in this study was the system developed by our research group capable of biasing the transistor with a constant current from 10 µA to 1 mA, with a resolution of 60 nA. It is also able to measure the source voltage, V_s_, with a resolution of 0.1 mV [21]. Let us remember that $V_{s}≅V_{T}$ in a saturated MOSFET under constant drain current biasing. In addition, it can be configured for continuous monitoring, providing the source voltage in real-time, biasing the transistor gate with an external voltage to improve the linearity and sensitivity. Moreover, device temperature can be monitored with the parasitic diode built in the MOSFET. To do that, a sensor module, composed of the MOSFET model 3N163 (Vishay, Malvern, PA, USA) and two JFETs model MMBF4391 (NXPSemiconductors, Eindhoven, The Netherlands) were designed. The JFET_GD connects and disconnects the MOSFET’s gate and drain terminals and JFET_SD the source and drain terminals. It can be configured in four states, controlled by voltages applied to the gate terminals of the JFETs acting as switches (see Figure 3):

- Storage mode: All the JFETs are ON (gates of JFETs are grounded), short circuiting all the terminals of the MOSFET to minimize gate oxide charge leakage during storage or between irradiations. The sensor module can be connected or not to the reader unit.
- Sensing mode: In this state, the gate and the drain of the MOSFET are disconnected (the JFET_GD is cut-off, applying a negative voltage in its gate, −13 V in our case) and the source and drain of the pMOS transistor are connected via JFET_SD. To increase the sensitivity, an external bias voltage is applied by the reader unit to the pMOS gate up to 25 V (0, 1, 5, and 10 V in this study).
- Readout of V_F_: In this state, both JFETs are cut off and the drain current is reversed to activate the parasitic diode, then the source voltage become negative, equal to less forward voltage of the parasitic diode (V_F_) [21]. This parameter is measured by the reader unit, and the die temperature is calculated from the linear relationship between the diode voltage and the temperature in a forwarded diode [25].
- Readout of V_S_: In this state the gate is connected to the drain (ground) via the JFET_GD, whose gate is grounded as well (see Figure 3 and Figure 4). The JFET_SD is cut-off, biasing its gate at negative voltage. In this configuration, the source is connected to the current source and the direct and amplified source voltage are measured, as well as the drain current by the reader unit after each irradiation.

**Figure 5:**
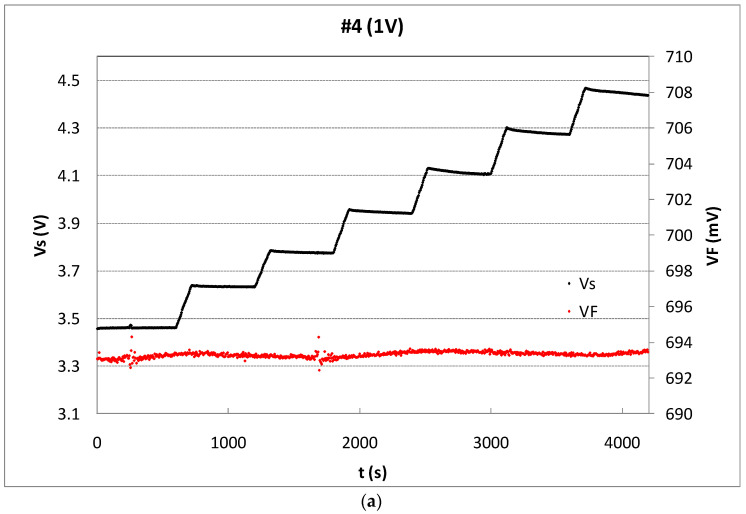

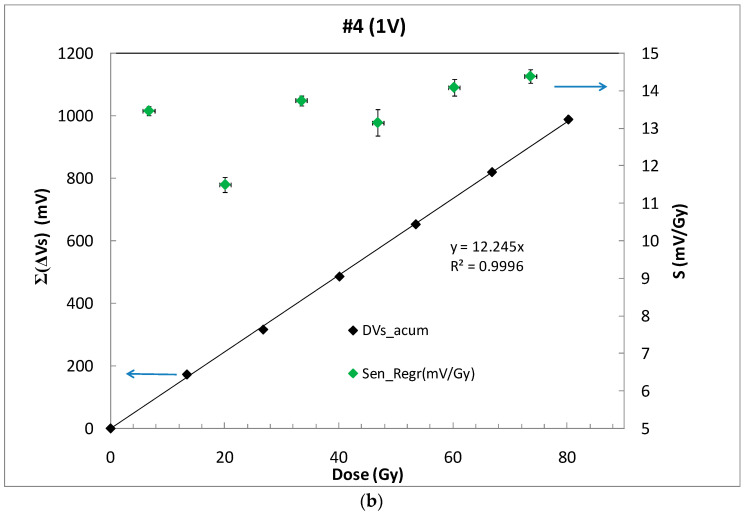
(**a**) Source voltage (black dots) of sample #4 biased by an external voltage of 1 V and V_F_ in mV (grey dots), during six irradiation shoots and rest periods. (**b**) Accumulated source voltage shift as a function of the accumulated dose (empty symbols) and line showing the least squared linear fitting. The sensitivity per irradiation shot is shown by solid symbols.

The readout of V_F_ or V_S_ and sensing modes are selected during each irradiation and rest periods and the results are sent to the computer every 4.6 seconds, as Figure 4 shows. To avoid the body effect, the bulk terminal is connected with the source terminal in all states. To minimize the temperature effect, the reader was configured to measure, biasing the pMOS in the readout mode at I_ZTC_, the minimum temperature coefficient drain current (230 µA in our case [26]), at this current the temperature coefficient of the source voltage is minimum. In addition, the silicon temperature was monitored during measurements, using the parasitic diode (see Figure 3 and Figure 5a). The V_F_ during the experiment showed a stable value, as Figure 5a shows, for the sample #4. The forward voltage of this sample was, in average, V_F_ = (693.38 ± 0.18) mV, that implies a temperature variation of approximately 0.1 ºC. The performance of the other samples was similar. Therefore, the temperature drift was not relevant during the irradiations in this study. Therefore, after each irradiation, in the readout mode, the complete measurement process consisted of three voltage and one current measurement. The first one was the forward voltage of the parasitic diode (V_F_), after the source voltage and the amplified source voltage (V_S_ and V_S,amp_, respectively), and finally the drain current (I_D_), as the oscilloscope screenshot shows in Figure 4, in which two complete irradiation-readout cycles are represented. The red and yellow lines show the gate-source voltages of the JFET_GD, V_G_JFET_GD_, and JFET_SD, V_G_JFET_SD_, respectively. The blue and green lines depict the gate-drain and source voltages, V_GD_ and V_S_, respectively.

## 3. Results and Discussion

The irradiation runs were planned in shots of 2 min providing a medium dose of 13.4 Gy for low values of bias voltages and a rest period of 8 min between shots. After a preliminary experimental study taking into account the sensitivity degradation of the sensor, the samples were irradiated until the source voltage was shifted 1 V. To ensure at least five irradiation shots per sample, without source shifts higher than 1V, the irradiation time was 1 min for the set biased at 10 V, 1:40 for the set biased at 5 V, and 2 min for 1 and 0 V, with a dose rate of 6.7 Gy/min. Under these experimental conditions, the number of shots for unbiased samples (0 V) was seven, and five for the samples biased at 10 V. Due to the high dose provided in each shot, and the sensor sensitivity, the source voltage without amplification, V_S_, was enough to evaluate the response of the transistors, as Figure 5a shows. In Figure 5b, the sensibility per shot was calculated considering a linear relationship between the source voltage shift, measured just at the beginning and the end of the shot, and the dose per shot (solid symbols). In this calculation, fading has not been account for and, thus, the MOSFET could be used as dose-rate dosimeter.

Alternatively, with empty symbols in Figure 5b, the sensitivity by shot has been calculated including the short-term fading effects, with the source voltage value not just after the irradiation but after the 8 min rest period between shots. Hereinafter, we will refer to this sensitivity as S. With these data, the average sensitivity was calculated as the slope of the accumulated source voltage shifts versus the accumulated dose. From Figure 5b, a high linear sensor response can be observed in terms of sensitivity.

**Figure 6:**
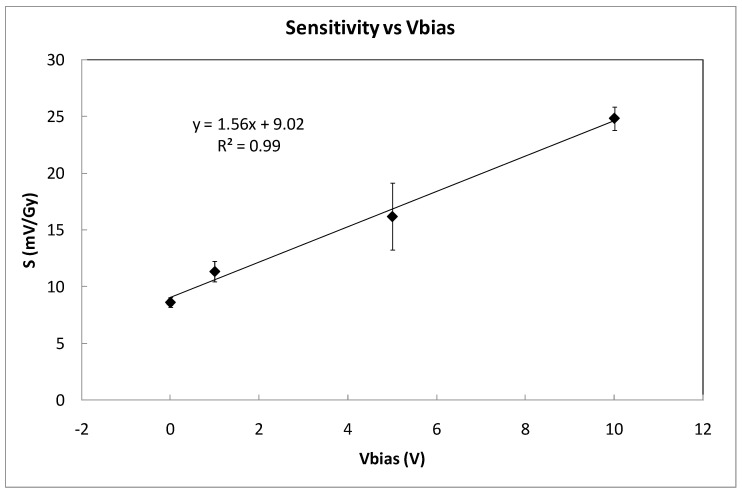
Average sensitivity as a function of the bias voltages (coverage factor k = 1). Symbols represent experimental data and the line indicates linear fitting.

**Figure 7:**
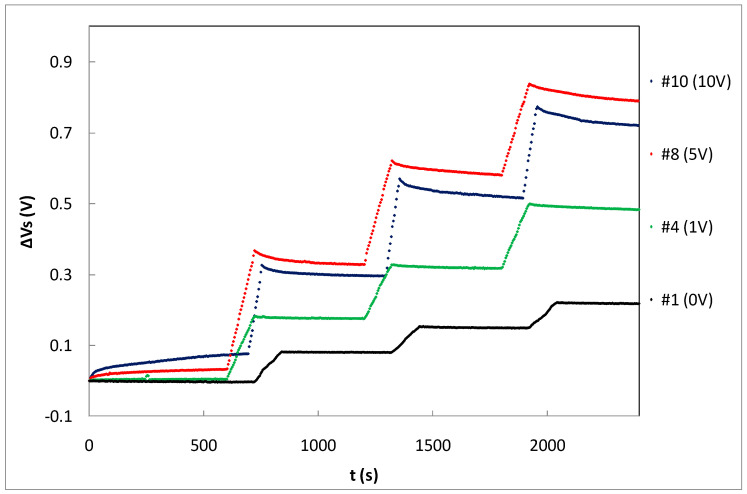
Source voltage increment during three irradiation shots of three samples biased at different gate voltages.

Table 1 summarizes the average sensitivity achieved with different bias voltages for individual samples and the average per set. Due to dispersion of the samples’ sensitivity, an individual calibration is required to be used as dosimeter. As was expected, the highest sensitivity was reached at the highest bias voltage due to the higher electric field inside the gate oxide that reduces the recombination ratio of the electron-hole pairs created by the ionizing radiation [14]. A linear dependence was found, showing a slope of $\left(1.56\pm 0.10\right)$ (mV/Gy)/V between the average sensitivity and the bias (gate) voltage as shown in Figure 6. According to this, it would seem that the most suitable voltage would be the highest studied value (10 V in our case). However, some factors should be considered as well. If the magnitude of interest is the dose-rate, determining the slope of the source voltage during irradiation (instant sensitivity) and high bias voltage would be more accurate. However, to calculate the accumulated dose, measuring before and after irradiations, the fading effect should be taken into account. In Figure 7, the source voltage shifts of four different samples biased at 0, 1, 5, and 10 V are displayed.

**Figure 8:**
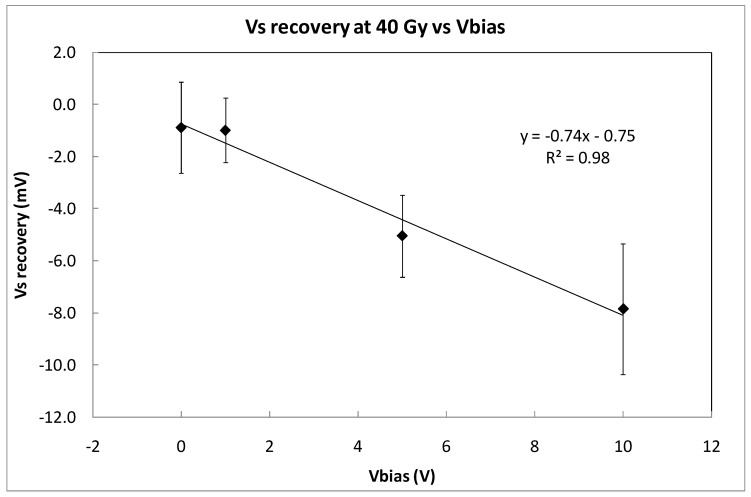
Fading quantified as the difference between the value of Vs at the end of the irradiation at 40 Gy and the value of the source voltage 5 min later. The coverage factor is k = 1. The symbols represents experimental data and the line indicates linear fitting.

To evaluate the short-fading effect, the recovery of Vs was measured five minutes after the third irradiation shot was concluded. Figure 8 shows the average recovery that was found for the four sets of three samples, biased at 0, 1, 5, and 10 V. As it was expected, the signal decay is higher at higher voltages, but very similar for 0 and 1 V. Taking into account the average sensitivity that was previously calculated at different bias voltages (Table 1), the dose error due to short fading effect can be calculated. The results are summarized in Table 2. Therefore, the bias voltage that was proposed to measure dose with this transistor could be 1 V due to an acceptable sensitivity value and the lowest dose error due to short-term fading effects.

In order to better understand these values for the sensor sensitivity, a comparative study of this parameter was carried out for the proposed pMOS 3N136 and other dosimetric MOSFETs. Table 3 shows these sensitivities for different irradiation conditions. From Table 3, we can observe a significant decrease of the sensitivity to protons compared with high-energy photons for the 3N163 sensor as it was previously pointed out for RADFETs [12,15]. This experimental response was explained because of an increased columnar recombination of the induced electron-hole pairs for the case of proton irradiation compared with ^60^Co, where geminate recombination dominates. Moreover, as was the case in this study, the columnar recombination is becoming even more important as the proton energy is decreased as the stopping power of protons decreases [13].

## 4. Conclusions

A characterization of the commercial pMOS transistor 3N163 (Vishay, USA) as proton dosimeter has been carried out. Due the low penetration power of the photons used in this study (5.36 MeV), the samples should be irradiated in a vacuum chamber. The modification of the samples by removing the top of the housing, was found as a suitable solution to avoid shielding of the protons by encapsulation. Under these experimental conditions, four external bias voltages of 0, 1, 5, and 10 V were tested, finding a linear dependence of the average sensitivity with the external bias voltage. However, at high bias voltage, the short fading effect, at 40 Gy, became more important, as it was expected. The bias voltage that presented a low fading effect with an acceptable sensitivity, (11.4 ± 0.9) mV/Gy, was 1 V. Moreover, the decrease of the sensitivity to the protons beam compared with high-energy photons can be explained based on the decreased stopping power of the proton energy. Therefore, the model 3N163 biased at 1 V shows some characteristics that made it a promising candidate as a proton dosimeter.

## Figures and Tables

**Table 1:** Sensitivity per sample and average sensitivity at different bias voltage.

MOSFET	S (Mv/Gy)	σ (S) (mV/Gy)	V_G_ (V)	Avg_S (mV/Gy)	σ (AvgSen) (mV/Gy)
#1	8.18	0.24	0	8.6	0.4
#2	8.66	0.15			
#3	9.08	0.09			
#4	12.24	0.05	1	11.4	0.9
#5	11.31	0.09			
#6	10.50	0.30			
#7	19.30	0.40	5	16.2	3.0
#8	13.40	0.40			
#9	15.81	0.23			
#10	23.60	0.50	10	24.8	1.0
#11	25.50	0.05			
#12	25.30	0.30			

**Table 2:** Error dose due to the fading effect.

V Bias (V)	Fading Dose Error (Gy)
0	−0.1 ± 0.2
1	−0.09 ± 0.11
5	−0.31 ± 0.11
10	−0.32 ± 0.10

Measurements taken after 40 Gy of accumulated dose, and 5 min after the irradiation ends.

**Table 3:** Response of different MOSFET used as proton dosimeters.

Source	Sensor	V Bias (V)	Sensitivity (mV/Gy)	Ref.
^60^Co photons	3N163	0	24.4 ± 0.9	[20]
6 MV photons from LINAC	3N136	0	20.1 ± 0.8	[20]
15 MV MV photons from LINAC	3N136	1	26.4 ± 0.8	[21]
190 MeV proton beam	TN-502RD	15	~250	[16]
62 MeV proton beam	TN-502RD	15	~75	[27]
60 MeV proton beam	Tyndall ESAPMOS4	0	~50	[15]
60 MeV proton beam	Tyndall ESAPMOS4	5	~130	[15]
5.36 MeV proton beam	3N163	1	11.4 ± 0.9	This work

## Data Availability

The data presented in this study are available on request from the corresponding author. The data are not publicly available due to the amount of data.

## References

[1] Steneker M., Lomax A., Schneider U. (2006). Intensity modulated photon and proton therapy for the treatment of head and neck tumors. Radiother. Oncol..

[2] Wilson V.C., McDonough J., Tochner Z. (2005). Proton beam irradiation in pediatric oncology: An overview. J. Pediatr. Hematol. Oncol..

[3] Haberer T., Debus J., Eickhoff H., Jäkel O., Schulz-Ertner D., Weber U. (2004). The heidelberg ion therapy center. Radiother. Oncol..

[4] Pedroni E., Scheib S., Böhringer T., Coray A., Grossmann M., Lin S., Lomax A. (2005). Experimental characterization and physical modelling of the dose distribution of scanned proton pencil beams. Phys. Med. Biol..

[5] Lomax A. (1999). Intensity modulation methods for proton radiotherapy. Phys. Med. Biol..

[6] Fontenot J.D., Lee A.K., Newhauser W.D. (2009). Risk of secondary malignant neoplasms from proton therapy and intensity-modulated x-ray therapy for early-stage prostate cancer. Int. J. Radiat. Oncol. Biol. Phys..

[7] Pedroni E., Bacher R., Blattmann H., Böhringer T., Coray A., Lomax A., Lin S., Munkel G., Scheib S., Schneider U. (1995). The 200-MeV proton therapy project at the Paul Scherrer Institute: Conceptual design and practical realization. Med. Phys..

[8] International Commission on Radiation Units and Measurements (1976). Determination of Absorbed Dose in a Patient Irradiated by Beams of X or Gamma Rays in Radiotherapy Procedures.

[9] Sorriaux J., Testa M., Paganetti H., Bertrand D., Lee J.A., Palmans H., Vynckier S., Sterpin E. (2017). Consistency in quality correction factors for ionization chamber dosimetry in scanned proton beam therapy. Med. Phys..

[10] Sowa U., Nowak T., Michalec B., Mierzwinska G., Swakoń J., Olko P. (2012). Dosimetric characteristics of active solid state detectors in a 60 MeV proton radiotherapy beam. Nukleonika.

[11] Cheng C.-W., Wolanski M., Zhao Q., Fanelli L., Gautam A., Pack D., Das I.J. (2010). Dosimetric characteristics of a single use MOSFET dosimeter for in vivo dosimetry in proton therapy. Med. Phys..

[12] Pease R.L., Simons M., Marshall P. (2001). Comparison of pMOSFET total dose response for Co-60 gammas and high-energy protons. IEEE Trans. Nucl. Sci..

[13] Ionizing Radiation Effects in MOS Devices and Circuits|Wiley. https://www.wiley.com/en-es/Ionizing+Radiation+Effects+in+MOS+Devices+and+Circuits-p-9780471848936.

[14] Holmes-Siedle A., Adams L. (1986). RADFET: A review of the use of metal-oxide-silicon devices as integrating dosimeters. Int. J. Radiat. Appl. Instrumentation. Part C. Radiat. Phys. Chem..

[15] Jaksic A., Kimoto Y., Mohammadzadeh A., Hajdas W. (2006). RADFET Response to Proton Irradiation Under Different Biasing Configurations. IEEE Trans. Nucl. Sci..

[16] Kohno R., Nishio T., Miyagishi T., Hirano E., Hotta K., Kawashima M., Ogino T. (2006). Experimental evaluation of a MOSFET dosimeter for proton dose measurements. Phys. Med. Biol..

[17] Kohno R., Hotta K., Matsubara K., Nishioka S., Matsuura T., Kawashima M. (2012). In vivo proton dosimetry using a MOSFET detector in an anthropomorphic phantom with tissue inhomogeneity. J. Appl. Clin. Med. Phys..

[18] Asensio L.J., Carvajal M.A., López-Villanueva J.A., Vilches M., Lallena A.M., Palma A.J. (2006). Evaluation of a low-cost commercial mosfet as radiation dosimeter. Sens. Actuators A Phys..

[19] Martínez-García M.S., Simancas F., Palma A.J., Lallena A.M., Banqueri J., Carvajal M.A. (2014). General purpose MOSFETs for the dosimetry of electron beams used in intra-operative radiotherapy. Sens. Actuators A Phys..

[20] Carvajal M.A., Simancas F., Guirado D., Vilches M., Lallena A.M., Palma A.J. (2012). A compact and low cost dosimetry system based on MOSFET for in vivo radiotherapy. Sens. Actuators A Phys..

[21] Carvajal M.A., Martínez-García M.S., Guirado D., Banqueri J., Palma A.J. (2016). Dose verification system based on MOS transistor for real-time measurement. Sens. Actuators A Phys..

[22] Carvajal M.A., Martínez-García M.S., Guirado D., Martínez-Olmos A., Palma A.J. (2016). Thermal compensation technique using the parasitic diode for DMOS transistors. Sens. Actuators A Phys..

[23] Cazzaniga C., García Alía R., Coronetti A., Bilko K., Morilla Y., Martín-Holgado P., Kastriotou M., Frost C.D. (2021). Measurements of Low-Energy Protons Using a Silicon Detector for Application to SEE Testing. IEEE Trans. Nucl. Sci..

[24] https://physics.nist.gov/PhysRefData/Star/Text/PSTAR.html.

[25] Fraden—Handbook-of-Modern-Sensors—Fraden—Handbook-of-Modern-Sensors|Docsity. https://www.docsity.com/pt/fraden-handbook-of-modern-sensors/4841727/.

[26] Carvajal M., Martínez Olmos A., Morales D., López-Villanueva J.A., Lallena A., Palma A. (2011). Thermal drift reduction with multiple bias current for MOSFET dosimeters. Phys. Med. Biol..

[27] Pablo Cirrone G.A., Cuttone G., Lojacono P.A., Nigro S.L., Patti I.V., Pittera S., Raffaele L., Sabini M.G., Salamone V., Valastro L.M. (2006). Preliminary investigation on the use of the MOSFET dosimeter in proton beams. Phys. Med..

